# Bone Marrow as a Source of DNA in Forensic Genetics: An Optimized Nucleic Acids Extraction Protocol

**DOI:** 10.3390/genes17030332

**Published:** 2026-03-18

**Authors:** Mattia Porcu, Noemi Argirò, Venusia Cortellini, Antonio De Luca, Camilla Tettamanti, Lorenzo Franceschetti, Francesco Ventura, Andrea Verzeletti

**Affiliations:** 1 Institute of Legal Medicine, University of Brescia, 25123 Brescia, Italy; m.porcu001@unibs.it (M.P.); noemi.argiro@unibs.it (N.A.); antonio.deluca@unibs.it (A.D.L.); andrea.verzeletti@unibs.it (A.V.); 2 Azienda Sociosanitaria Ligure ASL3, 16121 Genoa, Italy; camilla.tettamanti85@gmail.com; 3 Institute of Legal Medicine Milan, Department of Biomedical Sciences for Health, University of Milan, 20133 Milan, Italy; lorenzo.franceschetti@unimi.it; 4 Section of Legal and Forensic Medicine, Department of Health Sciences, University of Genoa, 16132 Genoa, Italy; francesco.ventura@unige.it; 5 IRCCS San Martino Hospital, 16132 Genoa, Italy

**Keywords:** bone marrow, DNA extraction, adipose tissue, forensic genetics, lipids putrefaction

## Abstract

Background: low-quantity or degraded samples are often studied in forensic genetics. Therefore, it is important to efficiently obtain all the available DNA from the biological sample analyzed to provide the most reliable results. This is particularly challenging in bone marrow processing due to its hydrophobic molecular structure, as for other lipid-rich tissues, especially if rancid. In fact, during adipose tissue decomposition, the putrefaction of fatty acids can in some instances give a compact cerous consistency to the lipidic tissue, hardly susceptible to the nucleic acid extraction mechanisms. According to environmental circumstances, this condition is notably observable in submerged bodies or in putrefied bone marrow. Thus, this study is focused on developing an optimized nucleic acids extraction protocol for putrefied bone marrow. Methods: genetic analyses were performed on putrefied yellow bone marrow collected from 20 human femora recovered from bodies in different decomposition stages. The optimized method was developed by integrating additional steps, reagents and time intervals on a silica-based column commercial kit. This strategy was compared in DNA yield to a standard extraction protocol, represented by the same commercial kit, but following the manufacturer’s directions. Both these strategies were tested in nucleic acid isolation efficiency by performing DNA typing, including real-time PCR quantification, Short Tandem Repeats (STR) amplification and fragments analysis steps. The analytical parameters evaluated were allele count, DNA concentration (ng/µL) and Degradation Index (DI). Results: for allele count and DNA concentration parameters, the optimized protocol showed clear and significant qualitative and quantitative improvements compared with the standard protocol, supporting its potential applicability in forensic casework and laying the foundation for future studies. Conclusions: prior to appropriate laboratory internal validation, the optimized protocol can be used for tough lipid-rich tissues processing without the need to purchase a dedicated system and using a same commercial kit routinely adopted for other forensic genetics matrices.

## 1. Introduction

In many forensic scenarios, human identification has to be performed under complex circumstantial and technical conditions, particularly when dealing with remains rather than recent biological samples. This is commonly encountered in Disaster Victim Identification (DVI) operations, where bodies and remains may be fragmented or recovered after prolonged periods. This results in highly heterogeneous preservation states [1,2,3].

As a result, during DNA profiling analyses, postmortem biological samples frequently exhibit low DNA yield, molecular fragmentation and may contain inhibitory reaction agents such as humic acids, urea and lipidic derivatives. All these factors can lead to partial or unsuccessful genetic profiles [4,5], challenging forensic genetic investigations and directly affecting both the choice of the suitable biological matrix to analyze and the efficacy of downstream laboratory workflows. Therefore, different sampling strategies and DNA extraction methodologies could be adopted depending on the biological tissue type and whether the remains are fresh, partially decomposed or severely degraded [1,6]. When dealing with fresh or partially decomposed bodies, biological fluids (e.g., blood) and soft tissues (e.g., skeletal muscle and parenchymal organs such as liver) are often preferred for genetic analysis. These samples typically contain sufficient non-degraded DNA quantities that can be processed using rapid, standardized and cost-effective laboratory procedures [7]. However, soft tissues are more susceptible to autolysis and microbial putrefaction. Therefore, degradation can progress relatively rapidly with increasing postmortem interval and environmental exposure [7,8]. As a consequence, the quantity and quality of DNA recoverable from these matrices may decrease over time, limiting their use in advanced decomposition stages.

In cases involving severe decomposition, skeletonization or prolonged environmental exposure, forensic analysis may rely on more resistant biological substrates. Hard tissues, such as bones and teeth, are commonly selected because they preserve structural integrity for a longer period and provide physical and chemical protection against degradation. For this reason, skeletal elements frequently represent the most reliable source of DNA—or the only remaining one—in complex forensic scenarios, including DVI operations and the recovery of long-decomposed human remains [9,10].

### 1.1. Bone Marrow as Source of DNA

Although hard tissues are among the most widely used substrates in forensic casework, other matrices associated with the skeletal system may also represent valuable sources of DNA [1,10]. Among these, bone marrow drew interest due to its anatomical location within the bone medullary cavity and its cellular composition [1,11]. Being enclosed within the bone, bone marrow is relatively protected from environmental exposure, which may contribute to its preservation, even under adverse postmortem conditions [12].

Bone marrow, classified as red or yellow, contains a high proportion of nucleated hematopoietic cells and adipocytes [11]. This characteristic makes it a potentially rich source of DNA when recoverable. Several studies have demonstrated that bone marrow can yield an amplifiable DNA suitable for genetic analysis, even when other biological matrices are limited or compromised [13,14]. In some forensic instances, bone marrow could be the only biological tissue available for DNA typing, as in the case of specific biopsy samples or paraffin-embedded tissues. For these reasons, bone marrow has been evaluated as an alternative or complementary substrate in genetic investigations, with particular attention to decomposed marrow for testing DNA profiling procedures in challenging forensic samples.

The forensic relevance of bone marrow has also been explored in disaster-victim identification contexts. In DVI operations, where different biological matrices may be available from the same individual, bone marrow has been included by De Boer et al. [1] among the tissues sampled for DNA analysis, alongside muscle, bone and teeth. These observations support the potential usefulness of bone marrow as a biological matrix for human identification, particularly in complex forensic contexts requiring flexible and case-dependent sampling strategies.

### 1.2. The Need for a Dedicated DNA Extraction Strategy

Despite its potential forensic genetics value, bone marrow presents specific analytical challenges, particularly due to its lipid-rich composition. In adults, the bone medullary cavity is largely occupied by yellow marrow, which predominantly consists of adipose tissue [11]. Lipid-rich biological matrices are widely recognized as challenging substrates for nucleic acid extraction, as their high fat content can interfere with DNA-isolation chemistry. High lipid content can promote the co-extraction of contaminants and complicate DNA-purification steps by hindering the separation of aqueous phases. It can also negatively affect tissue homogenization or cell lysis [6,15,16]. Accordingly, similar difficulties can be expected when processing bone marrow for genetic analyses. Extraction methods optimized for mineralized, keratinized or low-lipid tissues may be suboptimal, especially for yellow bone marrow, underscoring the need to carefully select an appropriate DNA extraction strategy.

Currently, DNA isolation from lipid-rich tissues can be performed through dedicated commercial kits precisely designed for high-lipid processing. However, these systems can be low-sensitive for forensic use, expensive and often optimized only for this type of tissue. These aspects, together with the necessity to process multiple and different biological matrices, may limit their availability in forensic laboratories. An alternative can be represented by the classic phenol-chloroform DNA extraction, a cost-effective method that, however, requires multiple steps and the handling of chemicals which are hazardous for health and may introduce PCR inhibitors. In light of these considerations, having different DNA-extraction options and strategies for lipid-rich tissues, including bone marrow, may be useful, particularly if they are safer and more commonly available in most laboratories. In fact, studies on lipid-rich tissues have further demonstrated that DNA yield and purity may vary substantially depending on the extraction chemistry adopted, highlighting potential limitations of standard workflows when applied to fat-enriched substrates [16]. These limitations may persist even with modern DNA-extraction kits, as optimized extraction chemistries can be required to overcome PCR inhibition and improve DNA recovery in challenging samples [17,18].

### 1.3. Putrefied Bone Marrow Challenges

The aforementioned challenges are markedly exacerbated under postmortem conditions. During adipose tissue decomposition, lipid components undergo biochemical transformations that substantially alter tissue properties. Specifically, the hydrolysis of triglycerides leads to the formation of free fatty acids which, in moisture-rich or anaerobic environments, can undergo saponification. This process results in a consequent adipocere formation, a stable wax-like substance. Such conditions can be typically observed in submerged bodies, in remains characterized by extended postmortem intervals or subjected to adverse climatic conditions [19]. In bone marrow, unlike fresh adipose tissue, this condition is related to the formation of an extremely hydrophobic, cerous and poorly penetrable matrix. This further hinders mechanical homogenization and limits the effectiveness of standard lysis procedures, potentially decreasing DNA recovery [19].

Although these limitations arise primarily during the extraction phase, their effects become evident at the amplification level. In addition to the potentially low DNA yield issue, residual co-extracted substances may also be present. If not efficiently removed, co-extracted lipids and their degradation products can persist in the final DNA eluate and act as PCR inhibitors, particularly when processing putrefied bone marrow rather than fresh adipose tissues. As reviewed by Sidstedt et al. [20], such inhibitors primarily affect amplification kinetics and enzyme activity, either by competing with the DNA polymerase for active sites (e.g., through interaction with the enzyme’s catalytic active site, thereby hindering nucleotide incorporation) or by chelating magnesium ions essential for the reaction. In Short Tandem Repeats (STR) typing analyses, in combination with limited DNA recovery and molecular degradation, these mechanisms can ultimately lead to artifacts such as allele dropout, peak imbalance or complete amplification failure.

### 1.4. Study Aim

In light of the previous considerations, putrefied yellow bone marrow represents a biologically and physicochemically challenging substrate, combining advanced decomposition with extreme lipid enrichment. This intrinsic matrix complexity could constitute the main upstream source of analytical difficulties and directly impact DNA extraction efficiency. However, systematic optimization of nucleic acid extraction protocols specifically targeting putrefied bone marrow remains comparatively limited. On this basis, the aim of the present study is to develop and evaluate an optimized DNA-extraction protocol designed for putrefied yellow bone marrow in forensic contexts. By introducing targeted modifications to a silica-based and commonly used commercial DNA-extraction system, this research seeks to improve DNA recovery by studying STR typing performance in comparison with the standard manufacturer’s protocol. The qualitative and quantitative effectiveness of the optimized method was assessed in terms of DNA yield and allele count. These results provide potential methodological improvements in highly challenging postmortem contexts and a foundation for broader forensic applications.

## 2. Materials and Methods

According to the literature [21,22], the analytical workflow implemented for all samples followed the standard procedures adopted for forensic STR typing: samples acquiring/receiving, samples inspection, aliquot or trace sampling, pre-treatment when required, DNA extraction, DNA quantification, DNA amplification, fragments analysis, electropherogram generation, genetic profile interpretation and statistical analysis. These steps are thoroughly described in the following paragraphs.

### 2.1. Context Description

The samples selected derived from a coastal cemetery landslide event described by Tettamanti et al. [23]. In this DVI-like incident, several human dead bodies and skeletal remains in different decomposition stages and burial ages were involved, some of which reached the seawater below. In 2021, after the investigations of the landslide event and the recovery of remains involved, the retrieved biological material was transported and stored at −20 °C in the Institute of Legal Medicine of the IRCCS San Martino Hospital (Genoa, Italy). Four years later, after the investigations had been concluded, some of the remaining putrefied bone samples were inspected and selected for the present study, as described in the following paragraph. The selected specimens were then transported to the Institute of Legal Medicine of the University of Brescia (Brescia, Italy) by authorized study authors under cold chain conditions.

### 2.2. Sample Size and Selection

Considering the scenario provided, the sample size of the present study was determined by selecting the bones that were not completely skeletonized, in order to acquire the bone marrow that was still present inside the medullary cavity. With the purpose of keeping the study aim on yellow bone marrow processing, a total of 20 intact human femora containing putrefied yellow bone marrow were selected. The integrity of each femur was verified to minimize the possible risk of environmental and cross-contamination of the marrow contained in long bones, thereby increasing the likelihood that each sample could be attributed to a single individual.

In addition, the samples were selected to represent different putrefaction phases and burial age, including bodies or remains recovered from dryland and submerged conditions. This approach was adopted to maximize the diversity within the samples studied, in order to test the efficiency of the DNA-extraction protocol in question across a broader range of decomposition stages and taphonomic conditions. The complete list of the cadavers or remains selected, including their conditions and information available, is shown in Table 1. However, the precise burial age was not retrievable due to the limited cemeterial details. Nevertheless, a burial interval of approximately 120 years can be referred to the cadavers or remains involved in the landslide, starting from the early 1900s up to 2015–2020.

### 2.3. Sampling Step

**Figure 1:**
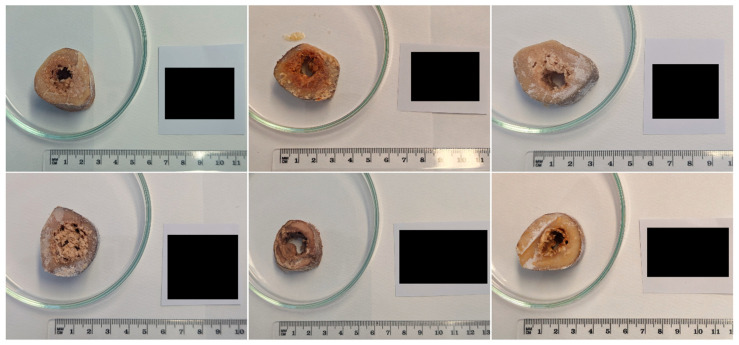
Examples of femoral diaphysis transversal sections obtained from the samples analyzed. Noting the decomposed bone marrow present in the bone medullary region.

According to the literature [24,25,26,27], the sampling step was performed on the femoral diaphysis middle portion of the selected long bones. It should be noted that the aforementioned references were used for pre-sampling procedures only, as they address bone tissue, whereas in the present study the target tissue was bone marrow. A transversal section about 5–10 cm long was obtained for each diaphysis sample using an oscillating necroscopy saw. Some examples are reported in Figure 1. Prior to this procedure, any residual soft tissue was removed, and the bone surface was decontaminated. The mechanical excision of the soft tissues, when present, was performed using scalpels and pliers decontaminated with sodium hypochlorite, while bone surface decontamination of the exposed femoral diaphysis was achieved through spraying 4% sodium hypochlorite, ultrapure water and absolute ethanol, in this precise order.

Although in the present study the target biological matrix was bone marrow rather than bone tissue, bone surface cleaning was performed to limit the environmental or cross-contamination events that could happen when sawing and potentially affect the inner bone marrow. Working surfaces and containers were decontaminated before and after handling each sample with 8% sodium hypochlorite solution, followed by washing with ultrapure water. Personal Protective Equipment (PPE) was correctly worn and changed between samples. Furthermore, the sampling room was isolated and reserved for this process.

After obtaining the femoral diaphysis section, 10 mg of putrefied yellow bone marrow were sampled and placed inside a 1.5 mL tube using hypochlorite-decontaminated scalpels, pliers and Pasteur pipettes. Since the study aim was to compare DNA-extraction efficiency between two methodologies, each bone marrow sample was collected in duplicate and transferred into two separate tubes for further processing.

To ensure an unbiased comparison between the two DNA-extraction protocols described in the following paragraph, the same conditions and procedures were performed for both the DNA-isolation strategies during the pre-extraction phases of the same specimen. Therefore, the yellow bone marrow was sampled from the same precise area for each duplicate aliquot deriving from a single marrow specimen. Overall, 20 bone marrow samples were processed with both extraction protocols (numerosity = 20 per protocol), for a total of 40 extracts submitted to DNA typing analyses.

### 2.4. DNA Extraction Protocols

Two DNA-extraction approaches were performed on the selected samples. The first is represented by a manufacturer’s extraction protocol provided with a QIAGEN^®^ (QIAGEN, Hilden, Germany) commercial kit commonly used in forensic genetics, whereas the second is an adaptation of the latter specifically designed for decomposed bone marrow or tough adipose tissues. This second optimized protocol was conceived considering the lipid-rich nature of bone marrow and the limited DNA yield achievable in some instances when using conventional nucleic acid isolation procedures on adipose tissues, as described in the introduction section.

In light of these considerations, the two DNA-extraction protocols were:

• Standard Extraction Protocol (SEP), represented by the “Isolation of Total DNA from Tissues” protocol, included in the “QIAamp^®^ DNA Investigator Kit” (QIAGEN, Hilden, Germany—Catalog Number: 56504). This commercial kit uses a silica-based membrane column to bind DNA. The system is specifically designed for forensic genetics purposes and is suitable for several tissues and substrates. The complete manufacturer’s protocol is available in the kit handbook [28] as well as in Supplementary Table S1;
• Optimized Extraction Protocol (OEP), conceived specifically for putrefied bone marrow or other tough lipidic tissues processing. This system was developed on the SEP: a pre-lysis phase was integrated, and the cell lysis step was modified, while the DNA isolation and purification approach was maintained unaltered. Therefore, as described in Table 2, the OEP starts from the biological material collection step, going through the pre-isolation phases (referred to as the “pre-lysis step” and “cell lysis step” in the OEP) and then resume the SEP at the point where the reagents required for the silica-membrane column DNA extraction are introduced (referred to as the “DNA isolation step” in the OEP).

In detail, the two extraction workflows shared the same commercial silica-based system but differed in the pre-lysis and lysis steps. The optimized extraction protocol (OEP) introduced (i) an additional pre-lysis treatment using a Sodium Dodecyl Sulfate (SDS) solution aimed at improving emulsification/homogenization of the lipid-rich matrix; (ii) prolonged digestion/lysis conditions, with overnight incubations performed under identical conditions (temperature and duration) for all samples; and (iii) specific buffer modification (ethylenediaminetetraacetic acid—EDTA and SDS solutions) to enhance cell disruption and reduce inhibitor carryover. In contrast, the standard extraction protocol (SEP) followed the manufacturer’s instructions without these modifications.

According to the sampling step, for each of the 20 bone marrow specimens the duplicate aliquots collected were processed using the two different extraction strategies described, one adopting the OEP and the other one using the SEP. In consideration of the putrefaction stages of the analyzed samples, for both protocols the DNA was eluted in a final volume of 40 µL. A negative extraction control (extraction blank) was included in each extraction batch and carried through quantification and STR amplification to monitor potential contamination.

### 2.5. DNA Quantification

Quantification of human DNA was performed on a “QuantStudio™ 5 Real-Time PCR Instrument” (Thermo Fisher Scientific, Waltham, MA, USA—Catalog Number: A28574), using the “Quantifiler™ Trio DNA Quantification Kit” (Thermo Fisher Scientific, Waltham, MA, USA—Catalog Number: 4482910) and following the manufacturer’s directions [29]. This commercial kit includes an internal PCR control (IPC) for each sample analyzed, which is used to assess PCR inhibition as indicated in the manufacturer’s manual [29]. Inhibition could be due to agents such as, for example, humic acids from soil or directly the putrefied lipids deriving from bone marrow. In order to evaluate this issue, for samples containing less than 5 ng/µL of human DNA and considering the IPC target threshold of 0.1, an IPC threshold cycle (C_T_) of 26–29 corresponded to no reaction inhibition, of 29–31 a limited inhibition and over 31 (or not detectable C_T_) a consistent inhibition, according to internal laboratory validations. In addition, each run included negative and positive controls, as well as duplicates of each DNA quantification standard sample needed per reaction plate. According to internal laboratory validations, the Limit of Detection (LoD) of the system adopted corresponded to 0.001 ng/µL of human DNA, and the upper limit of the standard curve was set at 100 ng/µL.

### 2.6. PCR Amplification

Human STR amplification was performed on a “Veriti™ 96-Well Thermal Cycler” (Thermo Fisher Scientific, Waltham, MA, USA—Catalog Number: 4375305) using the “PowerPlex^®^ Fusion 6C System” (Promega Corporation, Madison, WI, USA—Catalog Number: DC2705) and the “GlobalFiler™ IQC PCR Amplification Kit” (Thermo Fisher Scientific, Waltham, MA, USA—Catalog Number: A43565) according to the manufacturer’s directives [30,31]. The target DNA quantities adopted in PCR were determined based on the dilution recommendations of the quantification summary, in accordance with the reported “small probe” reagent values (see [29] for insights). As for the quantification step, each run included negative and positive PCR controls.

### 2.7. Fragments Analysis

Amplicons and allelic ladders were loaded on an “Applied Biosystems™ 3500 Genetic Analyzer” (Thermo Fisher Scientific, Waltham, MA, USA—Catalog Number: A43565) following the guidance of the respective PCR commercial kit. A 50 cm capillary array and POP-7™ polymer (Thermo Fisher Scientific, Waltham, MA, USA—Catalog Number: A26073) were used during fragments analysis. The run parameters for “GlobalFiler™ IQC PCR Amplification Kit” were set to 10 s injection time, 1.6 kV injection voltage, 15 kV pre-run voltage, 19.5 kV run voltage, 180 s pre-run time, 1330 s run time and 60 °C oven temperature. For “PowerPlex^®^ Fusion 6C System”, run parameters were set to 10 s injection time, 1.2 kV injection voltage, 15 kV pre-run voltage, 13 kV run voltage, 180 s pre-run time, 2000 s run time and 60 °C oven temperature.

### 2.8. Electropherogram Elaboration and Genetic Profile Generation

Allele calling of the raw data derived from the fragments-analysis step was achieved through “GeneMapper™ ID-X software v1.6” (Thermo Fisher Scientific, Waltham, MA, USA—Catalog Number: A38440). To evaluate the single-contributor nature of the analyzed samples and to assess potential amplification artifacts, the interpretation thresholds were set as follows: analytical threshold of 100 relative fluorescence units (RFU), stochastic threshold of 200 RFU, stutter threshold of 15%, peak height ratio of 50% and limit of linearity at 20,000 RFU. These parameters’ values were designed according to the literature [32] and internal laboratory validation.

In each of the 40 samples analyzed, the alleles called for the amplification systems used were unified in a consensus profile. According to the literature [33], consensus profiles were obtained by retaining only the alleles consistently observed across the PCR replicates for each STR locus shared between the amplification kits.

### 2.9. Study Endpoints

Three analytical parameters were evaluated for each sample analyzed: DNA concentration (ng/µL), Degradation Index (DI) and allele count. DNA concentration was directly obtained from the real-time PCR output and referred to the values reported by the “small probe” reagent provided by the “Quantifiler™ Trio DNA Quantification Kit”. Whenever a sample contained a concentration of human DNA not detectable (i.e., below the LoD), the assigned value corresponded to 0.000. DI, an adimensional qualitative indicator useful to assess DNA integrity, was automatically calculated by the quantification system and reported in the real-time PCR output (see [29] for insights). Allele count, a qualitative parameter also used for the assessment of DNA degradation degree and allelic drop-out [34], was determined manually by counting the alleles called on the consensus profiles generated for each sample analyzed. Considering the 21 autosomal STR loci shared between the two amplification kits, a complete profile should include 42 alleles. Thus, partial or null profiles ranged between 0 and 41 alleles. The complete list of shared STR markers evaluated in this study is reported in the Supplementary Table S2.

### 2.10. Statistical Evaluation

Data obtained from the analyses were statistically compared between the two extraction methodologies. For each bone marrow specimen, OEP and SEP results were paired to assess whether significant differences among means or medians were present for the 20 paired samples. Based on data distribution, DNA concentration and DI (non-parametric data) were evaluated using the Wilcoxon signed-rank test, whereas allele count (parametric data) was analyzed using the paired-samples *t*-test. All the statistical tests and descriptives were performed using jamovi software (Version 2.6) [35].

## 3. Results

**Figure 2:**
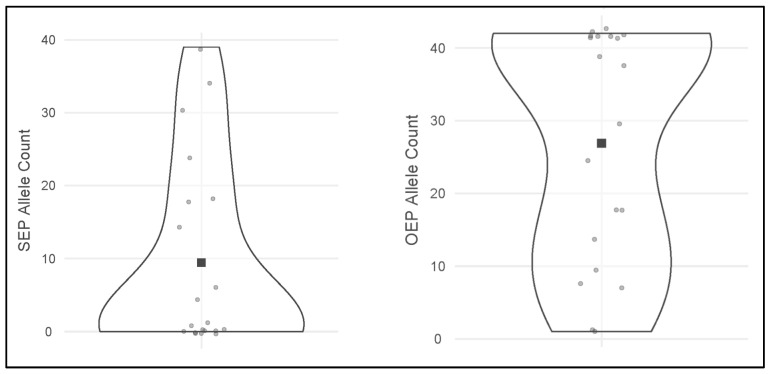
Distribution plots of the SEP allele count (**left**) and the OEP allele count (**right**) results. Each gray dot represents one of the samples analyzed, while the black square stands for the allele count mean. The violin representation visually indicates data distribution. SEP = Standard Extraction Protocol, OEP = Optimized Extraction Protocol.

**Figure 3:**
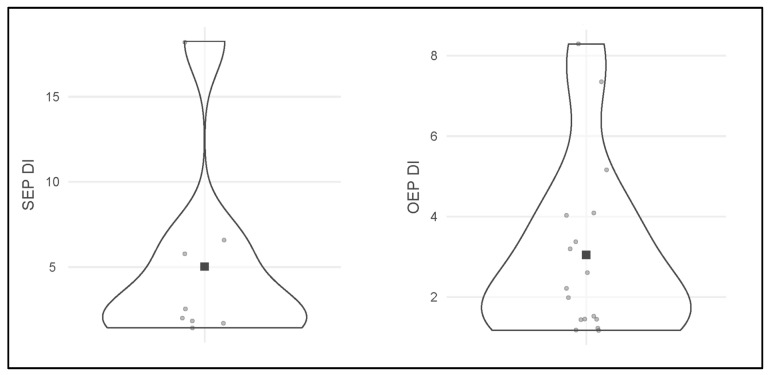
Distribution plots of the SEP DI (**left**) and the OEP DI (**right**) results. Each gray dot represents one of the samples analyzed, while the black square stands for the DI mean. The violin representation visually indicates data distribution. SEP = Standard Extraction Protocol, OEP = Optimized Extraction Protocol, DI = Degradation Index.

**Figure 4:**
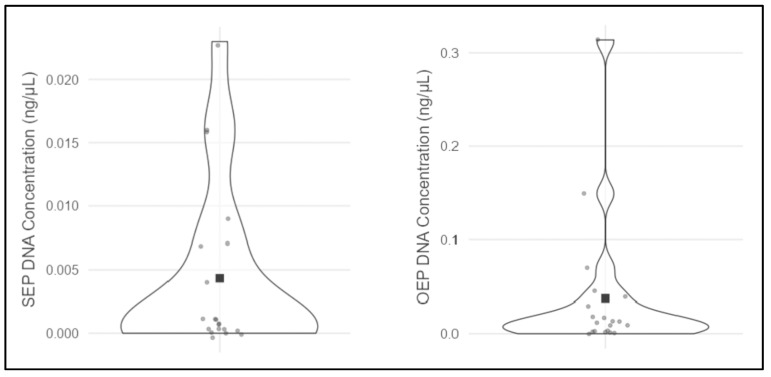
Distribution plots of the SEP DNA concentration (ng/µL) (**left**) and the OEP DNA concentration (ng/µL) (**right**) results. Each gray dot represents one of the samples analyzed, while the black square stands for the DNA concentration (ng/µL) mean. The violin representation visually indicates data distribution. SEP = Standard Extraction Protocol, OEP = Optimized Extraction Protocol.

The results for the 40 DNA-typing analyses, grouped in 20 paired samples for each evaluated endpoint, are reported in Table 3 and graphically shown in Figure 2, Figure 3 and Figure 4.

The electropherogram elaboration step indicated a single contributor for all the samples. Furthermore, for each extraction batch, real-time PCR and STR amplification, negative controls showed no amplification signal. Positive controls yielded the expected results in real-time PCR and the expected control DNA profile in STR amplification, as provided with the commercial kit. In addition, based on the real-time PCR report and amplification plots, the IPC C_T_ value for each sample was always included in a range of 26–29, which, in combination with the IPC curve shape evaluation, indicates the absence of relevant reaction inhibition.

**Figure 5:**
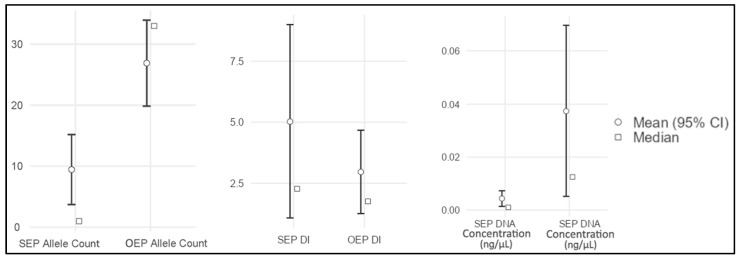
Mean and median plots of sample results, grouped by DNA extraction protocol (SEP or OEP) and endpoint considered (allele count—to the **left**; DI—in the **middle**; DNA concentration—to the **right**). SEP = Standard Extraction Protocol, OEP = Optimized Extraction Protocol, DI = Degradation Index, CI = confidence interval.

Descriptive statistics of the aforementioned results are reported in Table 4, and the corresponding plots are shown in Figure 5.

To assess data distribution, the Shapiro–Wilk normality test was performed for each analytical parameter. For both the extraction protocols, a non-parametric distribution was observed for DNA concentration (W-value = 0.54, *p*-value < 0.001) and DI endpoints results (W-value = 0.81, *p*-value = 0.039), whereas allele count showed a parametric distribution (W-value = 0.91, *p*-value = 0.063).

Accordingly, and as described in the Statistical evaluation paragraph, OEP and SEP paired samples were tested in extraction efficiency as reported in Table 5, in order to assess significant differences among means or medians of the analytical parameters studied.

Based on these results, significant differences were observed in allele count (paired samples *t*-test, *p* < 0.001) and DNA concentration (Wilcoxon signed-rank test, *p* = 0.003) in support of OEP. In contrast, no significant difference was observed for DI (Wilcoxon signed-rank test, *p* = 0.109) between the two extraction strategies.

## 4. Discussion

**Figure 6:**
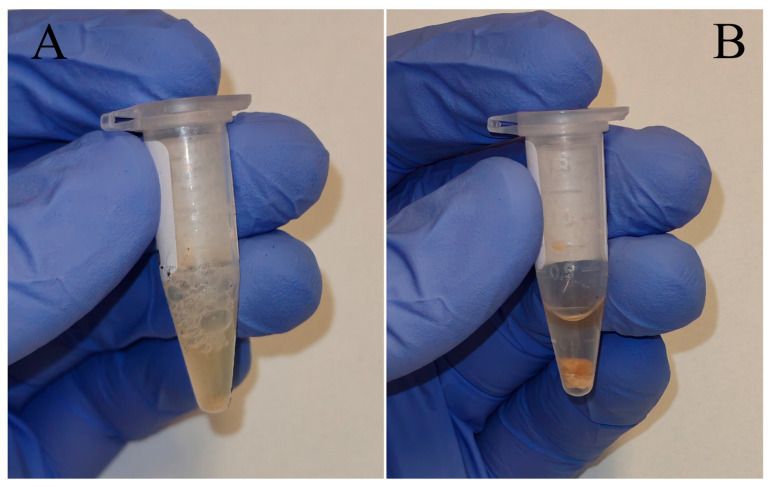
Example of the solutions obtained at the end of the lysis step (corresponding to OEP step 11 completion and SEP step 4 completion) of the same bone marrow specimen for the two DNA extraction protocols studied. (**A**): OEP post-lysis product, noting that solid bone marrow is not macroscopically present anymore. (**B**): SEP post-lysis product, noting that solid bone marrow is still present in the solution bottom. OEP = Optimized Extraction Protocol, SEP = Standard Extraction Protocol.

The better OEP endpoints values observed in the results underline the usefulness of the optimized protocol for processing putrefied bone marrow in forensic genetics. This outcome is most evident for allele count and DNA concentration parameters, two widely used indicators, for which the OEP yielded significantly higher values in most paired comparisons. These improvements could be justified by a more effective emulsification of the lipid-rich tissue during the OEP “Pre-lysis step” and “Cell lysis step”, presumedly achieved due to prolonged pre-extraction digestion and the use of surfactants such as SDS. Upon completion of this phase, differences in the degree of sample homogenization between the two protocols were mostly observed (Figure 6). Improved homogenization may facilitate cell disruption and the release of nucleic acids contained in bone marrow, thereby improving DNA recovery under OEP conditions. In addition, considering also the results observed through the real-time PCR inhibition assessment, the absence of inhibitors in the DNA extracts of each sample obtained from both the SEP and OEP strengthen the presumption that the better OEP DNA extraction efficiency arises likely due to the diverse quantities of successfully extracted DNA and its integrity, rather than a better inhibitors removal from the raw samples during extraction step.

Notably, in five out of 20 paired sample comparisons, the OEP provided a complete autosomal STR profile of 42 alleles (specimens 19, 43, 83, 211 and 227), whereas the SEP produced very limited profiles with less than five alleles called. For these samples, DNA concentration was at least 10–100-fold higher using the OEP than using the SEP.

Importantly, during SEP analyses, most of the DI values could not be included in the statistical evaluation because, for 12 specimens (samples 19, 43, 45, 83, 95, 96, 160, 171, 173, 211, 212A and 348), no signal was detectable and/or calculatable. This likely reflects the low-template DNA present in PCR reaction and its molecular degradation, which did not allow for the correct annealing between the real-time PCR probes and the target DNA (for insight see [29]). For these 12 SEP-processed samples, the “NO DATA” term was used as reported in Table 3 since, for DI, it is not possible to assign a fictitious numerical value as it ranges between 0 (which means fully intact DNA) and +∞. This aspect introduced an intrinsic issue in DI results interpretations; a non-detectable signal represents a negative outcome that is informative in itself, yet samples with missing values could not be considered in statistical calculations. As a consequence, the DI endpoint results should be considered with caution, independently from the eventual significant differences among the samples studied. In fact, for the present study, the authors decided to confer for both the SEP and OEP DI values a non-decisive weight-of-evidence, keeping them as just additional secondary information and basing the main study evaluation on allele count and DNA concentration only.

It is worth mentioning that, even during the OEP analysis, a few sample outcomes were labeled as “NO DATA” for the DI endpoint (samples 160, 173 and 348). However, in all these cases, the same lack of signal was also observed during the SEP analysis. Again, this suggests highly degraded and/or low-template samples, for which in this case even the OEP was not able to provide effective results for the DI parameter. Instead, in consideration of the DNA concentration endpoint, in one sample only (specimen 348) it was necessary to insert a value of 0.000 when using the OEP, while, for the SEP, seven samples reported a DNA concentration of 0.000 (specimens 83, 95, 96, 160, 211, 212A and 348).

In addition, another aspect to underline is the slightly better performance observed for the SEP in a few samples (samples 92, 116, 157 and 173). In these cases, allele count and/or DNA concentration parameters were equal or slightly higher in SEP than in OEP. This outcome could be linked to the intrinsic qualitative and quantitative characteristics of the endogenous nucleic acid present in bone marrow, which can lead to possible aliquot variability, especially in non-fresh specimens.

### 4.1. Limitations and Final Considerations

Although the OEP showed effective results, some limitations should be acknowledged. First, despite the taphonomic heterogeneity of the selected samples (see Table 1), all the cadavers or remains were derived from a single forensic context, potentially limiting the applicability of the results under different environmental and cadaveric conditions. Sample size should also be considered as a limitation (numerosity = 20), since the statistical evaluation could be partially distorted. Future studies should include more standardized taphonomic conditions and a larger sample size in order to strengthen the results and test the OEP across a broader range of specimens.

Another practical limitation is the additional overnight incubation required by the OEP, which increases processing time and may represent a restriction in forensic casework. Given this aspect, the OEP may be most appropriate for particularly challenging lipid-rich samples or situations in which standard protocols fail, while for routine or urgent casework laboratories should consider whether the additional time is acceptable for the case.

In light of all these considerations, and also concerning the DI parameter discussed above, statistical analyses still showed significant differences in allele count and DNA concentration between OEP and SEP, in favor of the optimized method. This confirms its adaptability for the selected samples across different decomposition stages. Conversely, differences in DI were not statistically significant between extraction strategies, in agreement with other literature reports [36]. This was not unexpected, given the limited reliability of DI in some contexts, particularly when DNA is neither fully intact nor completely degraded [37], as in the present case. For these aspects, the authors underline again the minor weight that should be attributed to DI in the interpretation of the present results.

### 4.2. Forensic Implementation and Future Perspectives

Beyond the analytical performance observed in the present study, practical and methodological considerations should be addressed to support the application of the proposed protocol.

Before routine implementation in forensic casework, the optimized protocol should be validated according to internal laboratory requirements. This validation should confirm that the workflow performs reliably across a wider range of postmortem conditions and sample variability, and that results are reproducible when the procedure is repeated by different operators and across different extraction batches. As part of this process, contamination control should be systematically monitored, for example by including extraction blanks, and PCR inhibition should be assessed using IPC-based evaluation.

Once properly validated, the OEP could theoretically also be adaptable to other commercial DNA-extraction kits and workflows, since the main modifications concern the pre-isolation step; however, this aspect requires dedicated evaluation. In addition, another relevant point concerns the downstream applications of DNA extracted using the OEP. In the present study, only STR typing was assessed, but the extracted DNA could also represent a suitable starting material for other forensic genetics applications, such as SNP typing, mitochondrial DNA analysis or Massive Parallel Sequencing (MPS), since the primary aim of the OEP is to maximize DNA recovery for subsequent analytical steps. These suppositions remain purely speculative, since they were not tested in the present study. Future studies should evaluate this aspect to provide solid evidence.

In addition to laboratory validation, further studies would be valuable to better define the practical role of bone marrow. An informative extension of the present work would be a direct, paired comparison between bone marrow and the corresponding bone matrix from the same individuals, to assess relative DNA yield and profiling success under comparable postmortem and environmental conditions. This paired study would allow a clearer assessment of whether bone marrow provides advantages over bone matrix (or the opposite) in advanced decomposition and would help define sampling recommendations for casework and DVI-like scenarios. While this comparison is outside the scope of the present manuscript, related bone-based analyses have been addressed in a previous work [36].

## 5. Conclusions

The DNA-extraction protocol developed for bone marrow processing proved to be effective, as the OEP showed clear and significant qualitative and quantitative improvements compared with the SEP. Accordingly, OEP could hypothetically be applied in real forensic genetics casework whenever bone marrow is involved. Nevertheless, the SEP naturally remains an excellent DNA-extraction strategy for more commonly processed tissues, along with the other protocols described in the “QIAamp^®^ DNA Investigator Kit”.

From a practical point of view, an advantage of the OEP is the possibility of processing tough lipid-rich tissues using the same commercial kit routinely adopted for other forensic genetics matrices, without the need to purchase a dedicated one. In that way, after appropriate internal validation, the OEP could be implemented within laboratory procedures based on the “QIAamp^®^ DNA Investigator Kit”, since the manufacturer currently does not provide a dedicated protocol for lipid-rich tissues.

In conclusion, the present work focused on putrefied yellow bone marrow and demonstrated the effectiveness of OEP in the context studied. This research lays the foundation for broader investigations to test the OEP efficiency for other decomposed lipid-rich tissues such as red bone marrow, subcutaneous adipose tissue, visceral adipose tissue and nervous tissue. Nevertheless, testing the OEP on fresh lipidic tissues should be accomplished to expand the potential range of applications.

## Figures and Tables

**Table 1:** Decomposition stages, taphonomic conditions and information available of the cadavers or remains selected for the present study.

Sample	Cadaver or Remains Decomposition Status	Sex	Estimated Age	Seawater Submersion (for Few Weeks)	Adipocere Presence	Bone Marrow Conditions
19	Liquefied-leathery mummified	Male	Elderly	Occurred	Not present	Abundant, solid
22	Liquefied	Female	Elderly	Not occurred	Not present	Abundant, Liquefied
24	Liquefied-partially skeletonized	Male	Elderly	Occurred	Not present	Scarce, solid
43	Liquefied-leathery mummified	Male	Adult	Occurred	Present	Abundant, liquefied
45	Liquefied	Female	Elderly	Occurred	Present	Abundant, Liquefied
83	Liquefied	Male	Adult	Occurred	Not present	Abundant, solid
90	Liquefied	Female	Elderly	Not occurred	Not present	Abundant, solid
92	Liquefied	Male	Elderly	Occurred	Present	Abundant, solid
95	Liquefied-partially skeletonized	Male	Adult	Occurred	Not present	Scarce, liquefied
96	Liquefied-partially mummified	Male	Elderly	Occurred	Not present	Scarce, liquefied
116	Liquefied-partially skeletonized	Male	Elderly	Occurred	Not present	Scarce, solid
157	Skeletonized	Female	Elderly	Not occurred	Not present	Scarce, solid
160	Skeletonized	Female	Elderly	Not occurred	Not present	Scarce, liquefied
171	Leathery mummified	Male	Elderly	Not occurred	Not present	Abundant, solid
173	Mummified	Female	Adult	Occurred	Not present	Scarce, solid
211	Liquefied-partially mummified	Male	Adult	Occurred	Present	Abundant, solid
212A	Mummified	Male	Elderly	Occurred	Not present	Scarce, solid
227	Mummified	Female	Elderly	Occurred	Not present	Scarce, solid
348	Liquefied	Male	Adult	Occurred	Present	Scarce, liquefied
351	Liquefied-partially mummified	Male	Elderly	Not occurred	Present	Scarce, solid

**Table 2:** Optimized Extraction Protocol (OEP) steps.

Pre-Lysis Step	Cell Lysis Step	DNA Isolation Step
1. In a 1.5 mL tube, place a maximum of 20 mg of bone marrow, ensuring that all the tissue reaches the bottom of the tube.	5. Centrifuge the tube at 8000 rpm for 15 s.	12. Refer to the “Isolation of Total DNA from Tissues” protocol” of the “QIAamp^®^ DNA Investigator Kit handbook” [28] (which represents the SEP) and proceed with the standard procedure resuming from adding 200 µL of AL buffer post-lysis step (corresponding to step 5 of the SEP) until the protocol end.
2. Add 50 µL of 20% SDS solution.	6. Add 180 µL of ATL buffer (provided in SEP kit).	
3. Mix thoroughly by vortexing.	7. Add 20 µL of Proteinase K (provided in SEP kit).	
4. Incubate the tube overnight in a thermomixer at 56 °C and 1000 rpm.	8. Add 15 µL of 0.5 M pH 8 EDTA solution.	
	9. Add 15 µL of 20% SDS solution.	
	10. Mix thoroughly by vortexing.	
	11. Incubate the tube overnight in a thermomixer at 56 °C and 1000 rpm.	

SDS = Sodium Dodecyl Sulfate, EDTA = Ethylenediaminetetraacetic acid, SEP = Standard Extraction Protocol.

**Table 3:** Results obtained in Standard Extraction Protocol (SEP) and Optimized Extraction Protocol (OEP) for DNA concentration (ng/µL), Degradation Index (DI) and allele count parameters, divided per sample analyzed. “NO DATA” mention refers to a not-detectable value (DNA concentration) or not-calculatable value (DI).

Sample	SEP DNA Concentration (ng/µL)	OEP DNA Concentration (ng/µL)	SEP DI	OEP DI	SEP Allele Count	OEP Allele Count
19	0.001	0.029	NO DATA	2.216	0	42
22	0.023	0.045	5.81	1.989	30	42
24	0.009	0.313	2.052	1.448	34	42
43	0.001	0.149	NO DATA	7.351	4	42
45	0.001	0.009	NO DATA	2.608	0	24
83	0.000	0.018	NO DATA	1.229	0	42
90	0.007	0.017	1.726	1.175	14	42
92	0.004	0.001	2.501	4.028	18	7
95	0.000	0.002	NO DATA	1.439	0	17
96	0.000	0.003	NO DATA	3.2	0	10
116	0.016	0.013	1.859	1.182	39	39
157	0.016	0.009	18.251	8.294	18	17
160	0.000	0.001	NO DATA	NO DATA	0	7
171	0.001	0.013	NO DATA	1.456	1	37
173	0.001	0.002	NO DATA	NO DATA	6	1
211	0.000	0.07	NO DATA	5.162	1	42
212A	0.000	0.003	NO DATA	3.372	0	13
227	0.001	0.039	1.449	1.529	0	42
348	0.000	0.000	NO DATA	NO DATA	0	1
351	0.007	0.012	6.573	4.09	24	29

**Table 4:** Descriptive statistics of samples results, grouped by DNA extraction protocol and endpoint considered.

Protocol and Endpoint	N	Mean	Median	SD	SE
SEP DNA concentration (ng/µL)	20	0.004	0.001	0.01	0.00
OEP DNA concentration (ng/µL)	20	0.04	0.01	0.07	0.02
SEP DI	8	5.03	2.28	5.69	2.01
OEP DI	17	3.05	2.22	2.16	0.87
SEP allele count	20	9.45	1.00	13.11	2.93
OEP allele count	20	26.90	33.00	16.05	3.59

SEP = Standard Extraction Protocol, OEP = Optimized Extraction Protocol, DI = Degradation Index, N = number of samples considered, SD = standard deviation, SE = standard error.

**Table 5:** Wilcoxon signed-rank test and/or paired samples *t*-test results obtained from the comparison between SEP and OEP for the means or medians of the analytical parameters studied.

Protocols Compared and Endpoint	Statistic	*p*-Value	Effect Size
SEP-OEP DNA concentration (ng/µL)	21.00 (W-value)	0.003 * (Wilcoxon signed-rank test)	−0.78
SEP-OEP DI	30.00 (W-value)	0.109 (Wilcoxon signed-rank test)	0.67
SEP-OEP allele count	−4.40 (Student’s t)	<0.001 * (paired samples *t*-test)	−0.98

* Significant *p*-value. SEP = Standard Extraction Protocol, OEP = Optimized Extraction Protocol, DI = Degradation Index.

## Data Availability

The original contributions presented in this study are included in the article/Supplementary Materials. Further inquiries can be directed to the corresponding author.

## Supplementary Materials

The following supporting information can be downloaded at: https://www.mdpi.com/article/10.3390/genes17030332/s1, Supplementary Table S1. “Isolation of Total DNA from Tissues” protocol defined in “QIAamp® DNA Investigator Kit” (QIAGEN, Hilden, Germany), representing the Standard Extraction Protocol (SEP). Supplementary Table S2. List of the STRs loci used for generation of the consensus profiles.
