# Supplementary material for: Bone Marrow as a Source of DNA in Forensic Genetics: An Optimized Nucleic Acids Extraction Protocol
Source: Genes (Basel). 2026 Mar 18;17(3):332. doi: 10.3390/genes17030332 (PMC13026300; doi:10.3390/genes17030332)
Supplement: Supplementary file 1 [file genes-17-00332-s001.zip › Supplementary Table S1.pdf]

**1.** Transfer a tissue sample of less than 10 mg in weight to a 1.5 ml microcentrifuge tube (not provided).
**2.** Immediately add 180 µl Buffer ATL, and equilibrate to room temperature (15–25°C).
**3.** Add 20 µl proteinase K and mix by pulse-vortexing for 15 s.
**4.** Place the 1.5 ml tube in a thermomixer or heated orbital incubator, and incubate at 56°C overnight or until the sample is completely lysed.<br>For small amounts of tissue, lysis is complete in 4–6 h, but best results are achieved after overnight lysis.
**5.** Add 200 µl Buffer AL, close the lid, and mix by pulse-vortexing for 15 s.<br>To ensure efficient lysis, it is essential that the sample and Buffer AL are thoroughly mixed to yield a homogeneous solution.<br>Note: If carrier RNA is required (see page 12), add 1 µg dissolved carrier RNA to 200 µl Buffer AL. Note that carrier RNA does not dissolve in Buffer AL. It must first be dissolved in Buffer ATE and then added to Buffer AL.
**6.** Add 200 µl ethanol (96–100%), close the lid, and mix thoroughly by pulse-vortexing for 15 s. Incubate for 5 min at room temperature.<br>Note: If room temperature exceeds 25°C, cool the ethanol on ice before adding to the tube.
**7.** Briefly centrifuge the 1.5 ml tube to remove drops from the inside of the lid.
**8.** Carefully transfer the entire lysate from step 7 to the QIAamp MinElute column (in a 2 ml collection tube) without wetting the rim, close the lid, and centrifuge at 6000 x g (8000 rpm) for 1 min. Place the QIAamp MinElute column in a clean 2 ml collection tube, and discard the collection tube containing the flow-through.<br>If the lysate has not completely passed through the membrane after centrifugation, centrifuge again at a higher speed until the QIAamp MinElute column is empty.
**9.** Carefully open the QIAamp MinElute column and add 500 µl Buffer AW1 without wetting the rim. Close the lid and centrifuge at 6000 x g (8000 rpm) for 1 min. Place the QIAamp MinElute column in a clean 2 ml collection tube, and discard the collection tube containing the flow-through.
**10.** Carefully open the QIAamp MinElute column and add 700 µl Buffer AW2 without wetting the rim. Close the lid and centrifuge at 6000 x g (8000 rpm) for 1 min. Place the QIAamp MinElute column in a clean 2 ml collection tube, and discard the collection tube containing the flow-through.<br>Contact between the QIAamp MinElute column and the flow-through should be avoided. Some centrifuge rotors may vibrate upon deceleration, resulting in the flow-through, which contains ethanol, coming into contact with the QIAamp MinElute column. Take care when

removing the QIAamp MinElute column and collection tube from the rotor, so that flow-through does not come into contact with the QIAamp MinElute column.
**11.** Carefully open the QIAamp MinElute column and add 700 µl of ethanol (96–100%) without wetting the rim. Close the cap and centrifuge at 6000 x g (8000 rpm) for 1 min. Place the QIAamp MinElute column in a clean 2 ml collection tube, and discard the collection tube containing the flow-through.
**12.** Centrifuge at full speed (20,000 x g; 14,000 rpm) for 3 min to dry the membrane completely.<br>This step is necessary, since ethanol carryover into the eluate may interfere with some downstream applications.
**13.** Place the QIAamp MinElute column in a clean 1.5 ml microcentrifuge tube (not provided), and discard the collection tube containing the flow-through. Carefully open the lid of the QIAamp MinElute column, and incubate at room temperature for 10 min or at 56°C for 3 min.
**14.** Apply 20–100 µl Buffer ATE or distilled water to the center of the membrane.<br>Important: Ensure that Buffer ATE or distilled water is equilibrated to room temperature. If using small elution volumes (<50 µl), dispense Buffer ATE or distilled water onto the center of the membrane to ensure complete elution of bound DNA.<br>QIAamp MinElute columns provide flexibility in the choice of elution volume. Choose a volume according to the requirements of the downstream application. Elution with small volumes increases the final DNA concentration in the eluate significantly, but reduces the overall DNA yield. Remember that the volume of eluate will be up to 5 µl less than the volume of elution solution applied to the column.
**15.** Close the lid and incubate at room temperature for 1 min. Centrifuge at full speed (20,000 x g; 14,000 rpm) for 1 min.<br>Incubating the QIAamp MinElute Column loaded with Buffer ATE or water for 5 min at room temperature before centrifugation generally increases DNA yield.

Supplementary Table S1. "*Isolation of Total DNA from Tissues*" protocol defined in "QIAamp® DNA Investigator Kit" (Qiagen, Hilden, Germany), representing the Standard Extraction Protocol (SEP).
The following procedure was retrieved without any modification from [QIAGEN (2020). QIAamp® DNA Investigator Handbook. Pages 52-55].
