# Supplementary material for: Bone Marrow as a Source of DNA in Forensic Genetics: An Optimized Nucleic Acids Extraction Protocol
Source: Genes (Basel). 2026 Mar 18;17(3):332. doi: 10.3390/genes17030332 (PMC13026300; doi:10.3390/genes17030332)
Supplement: Supplementary file 1 [file genes-17-00332-s001.zip › Supplementary Table S2.pdf]

STR locus	Chromosomal location
D3S1358	3p21.31
vWA	12p13.31
D16S539	16q24.1
CSF1PO	5q33.3-34
TPOX	2p23-2per
D8S1179	8q24.13
D21S11	21q11.2-q21
D18S51	18q21.33
D2S441	2p14
D19S433	19q12
TH01	11p15.5
FGA	4q28
D22S1045	22q12.3
D5S818	5q21-31
D13S317	13q22-31
D7S820	7q11.21-22
SE33	6q14
D10S1248	10q26.3
D1S1656	1q42.2
D12S391	12p13.2
D2S1338	2q35

Supplementary Table S2. List of the STRs loci used for generation of the consensus profiles. The chromosomal location was reported according to [Thermo Fisher Scientific (2025). GlobalFiler™ and GlobalFiler™ IQC PCR Amplification Kits USER GUIDE].
